# Surgical management of a huge mediastinal mature teratoma in a 2-year-old girl: a case report

**DOI:** 10.1186/s44215-024-00181-3

**Published:** 2024-12-20

**Authors:** Yusuke Matsui, Satoshi Shiono, Masahiro Mizumoto, Megumi Nakamura, Jun Suzuki, Hikaru Watanabe, Tetsuro Uchida

**Affiliations:** 1 Department of Surgery II, Faculty of Medicine, Yamagata University, 2-2-2 Iida-Nishi, Yamagata, Japan; 2 Department of Thoracic Surgery, Saitama Cardiovascular and Respiratory Center, Saitama, Japan

**Keywords:** Children, Mediastinal tumor, Mature teratoma, Surgery

## Abstract

**Background:**

Mature teratomas are benign cystic tumors that are most commonly asymptomatic. However, in some cases, mediastinal teratomas rupture the lungs and mediastinum with potentially fatal outcomes. Herein, we report a case of a large mediastinal mature teratoma that expanded to the entire left hemithorax in a child with common cold-like symptoms.

Case presentation.

A 2-year-7-month old girl visited a family doctor because of cough and rhinorrhea. Chest radiography revealed a large tumor occupying the left hemithorax, necessitating transfer to our institution. Chest computed tomography (CT) revealed a large tumor with calcifications and an encapsulated surface. The lesion was 10.5 cm in size and covered the entire left hemithorax, deviating significantly from the heart. CT suggested a mature mediastinal teratoma. Considering the risk of tumor dissemination, we did not perform a diagnostic biopsy; however, surgery was performed as an oncological emergency. As ventilation and circulation were difficult to maintain during the surgery, extracorporeal membrane oxygenation was performed. During surgery, although the large tumor tightly adhered to the sternum and innominate vein, it could be safely removed from these structures, and the tumor was completely removed through median sternotomy. The pathological diagnosis revealed a benign mature mediastinal teratoma. The patient’s postoperative course was uneventful.

**Conclusion:**

As the clinical course of child-specific problems in mature teratomas tends to be severe, a surgical strategy should be meticulously planned to ensure safety.

## Background

Mature teratomas are benign cystic tumors that account for approximately 60% of all mediastinal germ cell tumors [1]. More than 50% of mature mediastinal teratomas in adolescents are asymptomatic [2]; however, in some cases, these tumors can cause rupture of the lungs and mediastinum [3], which is clinically serious. In the case of a large mediastinal teratoma, preoperative planning of the optimal surgical strategy is essential because ventilation and circulation may be difficult to maintain during surgery.

In cases of mature mediastinal teratomas in children, differential diagnosis is sometimes difficult by physical examination because the symptoms are similar to common cold-like symptoms. Furthermore, mediastinal teratomas in children can result in serious conditions [2] owing to the child’s characteristics. Herein, we report a case of a large mediastinal mature teratoma that expanded to the entire left hemithorax in a child with common cold-like symptoms.

## Case presentation

The patient was a 2-year-7-month old girl who was healthy at birth. She initially visited a home doctor because of a cough and rhinorrhea. Although she was diagnosed with RS virus infection and received conservative treatment, the symptoms did not improve. She was referred to another hospital where chest radiography revealed a large tumor occupying the left hemithorax. She was diagnosed with a mediastinal tumor and admitted to the pediatric department of our institute. On physical examination, the patient appeared healthy and active, with no facial edema or engorgement of the neck vessels. Her height was 85 cm, weight was 11.7 kg, and temperature was 38.0 °C. The SpO_2_ was 98% in room air. Here, the pulse rate was 123/min, regular, and her regular respiratory rate was 30 breaths/min. The patient’s left breathing sounds were not auscultated. Blood analyses revealed an elevated white blood cell count (22,510/µL) and C-reactive protein level (3.06 mg/dL). Regarding tumor markers, alpha-fetoprotein was normal (2.6 ng/mL), but CA19-9 was high (2658 U/mL).

**Fig. 1:**
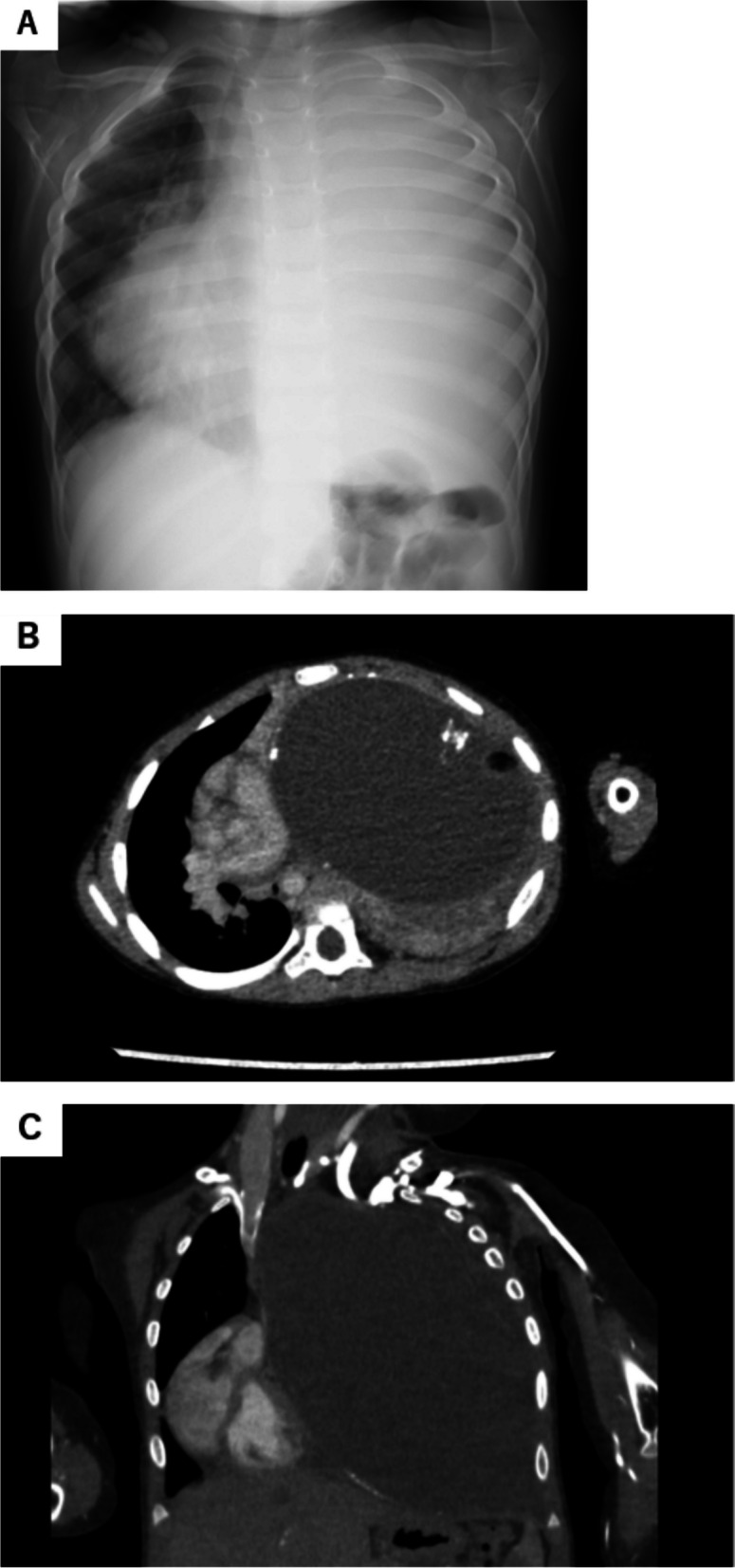
Chest radiograph (**A**) showing a huge tumor occupying the left thoracic cavity, and the heart shadow deviated to the right side. Chest CT showing a huge tumor, 10.5 cm in size. The tumor had calcified and adipose tissue components and completely occupied the left cavity. Images are shown from **B** axial and **C** coronal views

Chest radiography demonstrated that the tumor completely occupied the left hemithorax, and the mediastinum shifted to the right side (Fig. 1A). Chest computed tomography (CT) revealed a large tumor with calcification and an encapsulated surface 10.5 cm in size in the entire left hemithorax. The tumor significantly deviated from the heart. Owing to compression of the left main bronchus, the left lung showed complete atelectasis (Fig. 1B and C). Additionally, the tumor was cystic with adipose tissue and calcification, and a mature mediastinal teratoma was suspected. Transthoracic echocardiography revealed compression of the heart to the right side, diastolic dysfunction of the right ventricle, and expansion of the superior and inferior vena cava. Biopsy was not performed because of concerns regarding tumor dissemination.

**Fig. 2:**
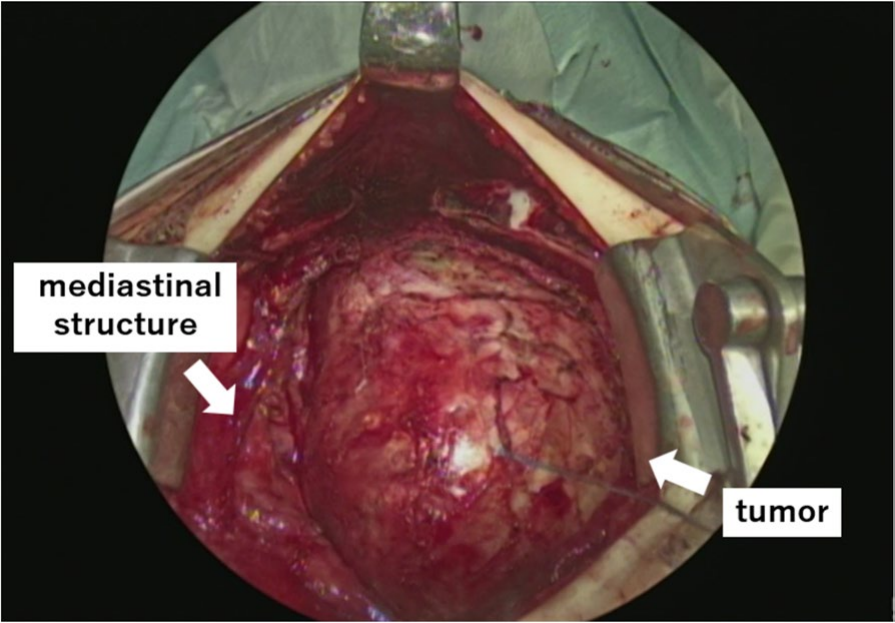
A tumor is seen in the midline, and the thymus and heart are compressed to the right side

Because the tumor significantly compressed the mediastinal structure, we decided to perform emergency surgery. We predicted that ventilation and circulation would be difficult to maintain during the induction of general anesthesia; therefore, extracorporeal membrane oxygenation (ECMO) was initiated. General anesthesia was induced in the left lateral recumbent position without muscle relaxation, and the patient was intubated using a single-lumen endotracheal tube. The patient was then placed in the supine position, but no particular change in his circulatory or respiratory status was observed. A median sternotomy was performed, and the tumor was located in the anterior mediastinum of the left thorax. The mediastinum, including the thymus and heart, was compressed on the right side (Fig. 2). Although it tightly adhered to the sternum and innominate vein, it could be safely removed from these structures. However, to ensure a safe surgical procedure, 450 mL of milky white content was aspirated prior to removal. Subsequently, the tumor was completely removed via median sternotomy. No tumor invasion of other organs was observed. Macroscopically, the tumor contained sebum, hair, and bone. Serum CA19-9 was elevated but there was no obvious pancreatic tissue. Frozen section diagnosis revealed a mature teratoma without immature tissue. Because the left lung had completely collapsed, we were concerned about the risk of re-expansion pulmonary edema; thus, the left lung slowly expanded, and the patient was administered preventive methylprednisolone. Two days after surgery, the patient was extubated and recovered smoothly. Because vital signs and ventilation were stable during surgery, we did not use ECMO.

**Fig. 3:**
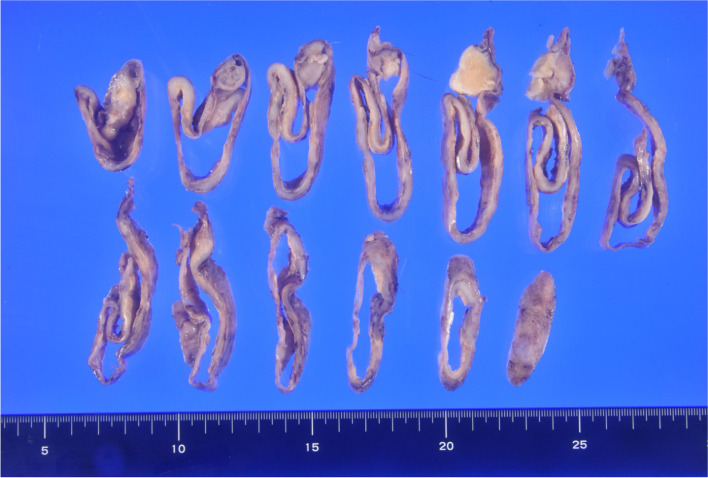
Macroscopic findings of cross-section of the tumor composed of hair, bone, and sebum

Macroscopic examination revealed that the tumor was 11.5 cm in size and contained hair, bone, and sebum (Fig. 3). The pathological diagnosis was a benign mature teratoma. The patient is doing well 1 year after surgery, with no signs of tumor recurrence.

## Discussion

Germ cell tumors are the third most common pediatric mediastinal tumors. Mature teratomas are benign cystic tumors that account for approximately 60% of all mediastinal germ cell tumors [1]. Although mature teratomas are benign, there have been several reports of large masses triggering serious symptoms in children [4, 5]. Compared with adult mediastinal teratoma cases, pediatric cases show different symptoms and diagnoses, and infants and children are more likely to develop symptoms than adults. There have been several suggestions to explain this [2]: (1) the frequency of malignant tumors in children is higher than that in adults, (2) the symptoms of compression or invasion of tumors are more likely to occur due to the small thoracic size, and (3) tumors are more commonly located close to the trachea. In particular, among all patients with respiratory distress, more than three-quarters of less than 2-year-old children had symptoms of tracheal compression [2]. We believe that this case report is instructive. The 2-year-old child did not report their own symptoms clearly, and the pediatrician could not think of taking a chest radiograph for the children suffering from cough.

Even in benign mature teratomas, serious symptoms can develop in pediatric cases. However, the performance of a diagnostic biopsy remains controversial. Nevertheless, there have been several reports on diagnostic biopsies [4, 5]. We did not perform a diagnostic biopsy for two reasons. First, sedation can cause catastrophic cardiopulmonary complications in children with respiratory distress. Although diagnostic biopsy for anterior mediastinal tumors in children is high-risk, Halepota et al. reviewed 35 children with anterior mediastinal masses who underwent diagnostic biopsy under general anesthesia and found no cases of on-table mortality [6]. However, they reported that two patients had laryngospasms and two had desaturation. Second, we were concerned about the risk of tumor dissemination if the tumor was malignant. Chang et al. reported a case of cystic malignant teratoma that recurred due to intraoperative spillage [7]. Although the contents of the tumor were aspirated in the present case to obtain a safe surgical view, as Yokoyama et al. reported [4], the tumor contents must be treated as carefully as possible.

In the present case, the radiological findings were characteristic of mature teratomas, including a well-demarcated shape and presence of adipose tissue, fluid, and calcification [1, 8]. According to a review by Ranganath et al., immature malignant teratomas tend to have a solid component more commonly than mature benign teratomas. Additionally, mature benign teratomas do not invade adjacent structures [1]. Considering risk management, a diagnostic biopsy is not mandatory if CT findings demonstrate the characteristics of a mediastinal tumor. In the present case, we did not perform magnetic resonance imaging (MRI) because it is time-consuming and requires sedation, which carries the potential risk of cardiopulmonary complications.

There have been reports of airway obstruction after general anesthesia or muscle relaxants in pediatric mediastinal tumors [9]. In this case, a muscle relaxant was not used for induction of anesthesia, and the patient was positioned in the left lateral recumbent position and then supine. ECMO was performed on a standby basis in cases of intraoperative cardiopulmonary complications. Yokoyama et al. also prepared ECMO for an 11-year-old female with a large mature mediastinal teratoma that occupied the entire left hemithorax [4]. Fortunately, ECMO was not performed in this case. Therefore, careful perioperative management is required.

## Conclusion

Herein, we report a case of a large mediastinal mature teratoma in a 2-year-old girl. As the clinical course of child-specific problems tends to be severe, a surgical strategy should be meticulously planned to ensure safe surgery.

## Data Availability

The authors have full control over all primary data and agree to allow the journal to review their data if requested.
